# Review of "Leishmania- *after the Genome*" by Peter J. Myler and Nicolas Fasel

**DOI:** 10.1186/1756-3305-1-11

**Published:** 2008-05-13

**Authors:** Kevin M Tyler

**Affiliations:** 1 BioMedical Research Centre, School of Medicine, Health Policy and Practice, University of East Anglia, Norwich, NR4 7TJ, UK

## Review

With the recent publication of complete genome sequences for *Leishmania major*, *Trypanosoma brucei *and *Trypanosoma cruzi *has come the perception of an opportunity to pause, survey and reflect upon the current state of research for these organisms. To consider the shape of things to come and, in particular, how best to modernize future studies by imbuing them with insights mined from the huge genomic and post-genomic datasets now available. Into this zeitgeist a number of books have already been launched starting before the genome publication with useful texts such as Melville's " Parasite Genomic Protocols" in 2004 and then subsequently Barry's "Trypanosomes: After the Genome" last year and perhaps a little more tangentially Olson and Bhattacharya's "The Genomics and Evolution of Microbial Eukaryotes " in 2006.

This is the second edited text devoted to *Leishmania *in the past few years. It does have a minor degree of overlap with the first, edited by Farrell in 2002, which was simply entitled "*Leishmania*" and was part of the "World Class Parasites Series". This somewhat weightier, stand-alone tome is updated and is a more unabashedly technical treatise than the first. The work is aimed as a reference for modern parasitologists working in *Leishmania *research and in allied fields. It will undoubtedly be a mandatory text for PhD students in contributing and affiliated laboratories. Indeed, the editors have done well in securing high quality contributions from most of the top leishmaniasis research laboratories in the world. The composition of the text is essentially an expert set of contemporary reviews which give a snapshot of research as it stands now, shortly after completion of the *Leishmania *genome. Many of the reviews have annotated large data sets into comprehensive tables and information-rich diagrams, which confirm its utility as a reference text and to a degree justifies its publication in book format and inclusion in medical reference libraries and collections. Indeed, these tables provide useful detail on everything from the enzymes of *Leishmania*'s peculiar metabolic pathways, to current vaccine candidates, to proteins implicated in drug resistance, to those with potential roles in vector interaction. The book itself is available as a modestly priced hardback. The cover art and finish is attractive but the content is black and white apart from two appendixed colour plates and the text is crowded into a two-column format which reduces its readability.

As an open access journal, *Parasites and Vectors *seems a reasonable place to raise the issue of the future of such texts. Leishmaniasis is considered a neglected disease and its major research funders such as the Wellcome Trust and the NIH are increasingly demanding that research funded by them is published in open access forums. Books such as this one remain an exception. This raises the issue of whether, in an age of open access, an alternative such as a "guest edited" special issue of an open access journal would be a better publishing option for such reviews. The benefits to authors are substantial in that they can increase the impact, influence and circulation of their articles, extending their reach worldwide to wherever the internet is available and particularly into the countries where the diseases are endemic. Further, and contrary to popular belief, electronic open access publication almost certainly extends the shelf-life of the articles, as print runs for such books tend to be of relatively short duration. I believe that the time for publishing reviews in book format is drawing to a close. I would, therefore, call on those who are considering compiling themed reviews in parasitology to work with us at *Parasites and Vectors *to provide suitable electronic, open access alternatives.

## Competing interests

The author declares that he is a member of the Advisory Board of *Parasites & Vectors*.

